# Pseudohypoparathyroidism Type 1A in an Omani Family Demonstrating Phenotypic Heterogeneity and Progressive Biochemical Changes

**DOI:** 10.7759/cureus.110964

**Published:** 2026-06-16

**Authors:** Taher A Al Shabibi, Maryam Al Badi, Wafa Abdelrahman

**Affiliations:** 1 Pediatrics, Ibra Hospital, Ibra, OMN; 2 Pediatric Endocrinology, National Diabetes and Endocrine Center, Royal Hospital, Muscat, OMN

**Keywords:** albright hereditary osteodystrophy, gnas mutation, hormone resistance, intrafamilial heterogeneity, osteoma cutis, pseudohypoparathyroidism type 1a

## Abstract

Pseudohypoparathyroidism type 1A (PHP1A) is an uncommon inherited condition caused by loss‑of‑function mutations in the guanine nucleotide-binding protein, alpha-stimulating activity polypeptide (GNAS) gene, which results in hormone resistance to parathyroid hormone (PTH) and other hormones that signal through the stimulatory alpha subunit of the G protein (Gsα). The disorder is classically associated with Albright hereditary osteodystrophy (AHO), although marked phenotypic and biochemical variability has been increasingly recognized, particularly in children.

We report three siblings from an Omani family diagnosed with PHP1A, aged 2.6-9 years, demonstrating substantial intrafamilial heterogeneity. The eldest sibling presented with severe multisystem involvement, including congenital aortic stenosis, extensive osteoma cutis, and neurocognitive impairment. The proband had a more classical endocrine phenotype with progressive PTH elevation and symptomatic soft tissue ossifications. The youngest sibling was identified through family screening during infancy and remained largely asymptomatic despite evolving biochemical abnormalities. All siblings demonstrated characteristic AHO features including round facies, brachydactyly, and shortening of the fourth and fifth metacarpals and metatarsals. Longitudinal biochemical evaluation showed progressive PTH elevation with persistent normocalcemia and hyperphosphatemia. Genetic testing in one sibling confirmed a heterozygous pathogenic GNAS mutation.

This familial case series highlights the broad phenotypic spectrum and dynamic biochemical evolution of PHP1A. AHO manifestations may precede overt biochemical abnormalities for several years, particularly during childhood. Recognition of subtle early features, proactive family screening, and longitudinal biochemical monitoring are essential for timely diagnosis and management.

## Introduction

Pseudohypoparathyroidism (PHP) comprises a heterogeneous group of disorders characterized by end‑organ resistance to parathyroid hormone (PTH) and abnormalities in G protein-coupled receptor signaling. PHP type 1A (PHP1A) results from maternally inherited inactivating mutations of the guanine nucleotide-binding protein, alpha-stimulating activity polypeptide (GNAS) gene on chromosome 20q13, which encodes the stimulatory alpha subunit of the G protein (Gsα) [1,2]. The condition is transmitted in an autosomal dominant manner with tissue‑specific genomic imprinting, producing variable hormone resistance across affected organs.

PHP1A is an ultra-rare disorder (estimated prevalence 0.3-1.1 per 100,000), with no available prevalence data from Oman or the Gulf region and only isolated reported cases.

Classically, PHP1A presents with hypocalcemia, hyperphosphatemia, elevated PTH, and the physical stigmata of Albright hereditary osteodystrophy (AHO), including round facies, obesity, brachydactyly, subcutaneous ossifications, short stature, and variable intellectual impairment [3]. Nevertheless, growing evidence indicates substantial variability in both clinical expression and biochemical evolution, particularly in pediatric cohorts; age at onset, the pattern and severity of hormone resistance, and neurocognitive outcomes can differ markedly between patients [4,5].

Despite a well‑defined molecular cause, important gaps remain in our understanding of intrafamilial phenotypic variability, progressive biochemical changes over time, and how modifying factors (age, imprinting effects, and environment) influence clinical trajectories, especially in underreported populations. Few series systematically document discordant endocrine phenotypes or progressive laboratory evolution among siblings who presumably share the same pathogenic GNAS variant. This lack of detailed familial data can delay recognition of nonclassic presentations and complicate genetic counseling and long‑term management.

We describe three siblings from a single Omani family, including one with genetically confirmed PHP1A, who demonstrate marked phenotypic heterogeneity and progressive biochemical evolution despite a presumed shared genetic background. By detailing their divergent ages at presentation, differing degrees of PTH resistance, and variable AHO and neurocognitive features, we aim to expand the recognized phenotype of PHP1A and to highlight diagnostic, counseling, and follow‑up implications for clinicians.

## Case presentation

Patient 1 (older brother)

A nine-year-old male patient initially presented at five years of age with multiple painful subcutaneous ossifications causing progressive limitation of joint mobility. Retrospective history revealed isolated ectopic calcifications beginning at approximately two years of age, followed by gradual dissemination over subsequent years.

His medical history was significant for critical congenital aortic stenosis requiring balloon valvuloplasty at 12 months of age and bronchial asthma. Clinical examination demonstrated characteristic AHO features, including round facies, central obesity, brachydactyly, and shortening of the fourth and fifth metacarpals and metatarsals. Height and weight were both at the 50th percentile according to the World Health Organization growth charts.

Neurocognitive evaluation revealed significant learning difficulties with a full-scale intelligence quotient (IQ) of 87 ± 5, requiring educational support.

Longitudinal biochemical assessment demonstrated progressive elevation of PTH levels up to 21 pmol/L, while serum calcium remained within the normal range. Serum phosphate levels were persistently elevated. The thyroid function test (TFT) showed fluctuating subclinical hypothyroidism.

Skin biopsy confirmed osteoma cutis. Cranial computed tomography showed no evidence of basal ganglia calcification. Echocardiography demonstrated stable post-valvuloplasty findings without residual calcification. Skeletal surveys revealed progressive ectopic calcifications involving multiple anatomical sites.

Patient 2 (proband)

**Figure 1:**
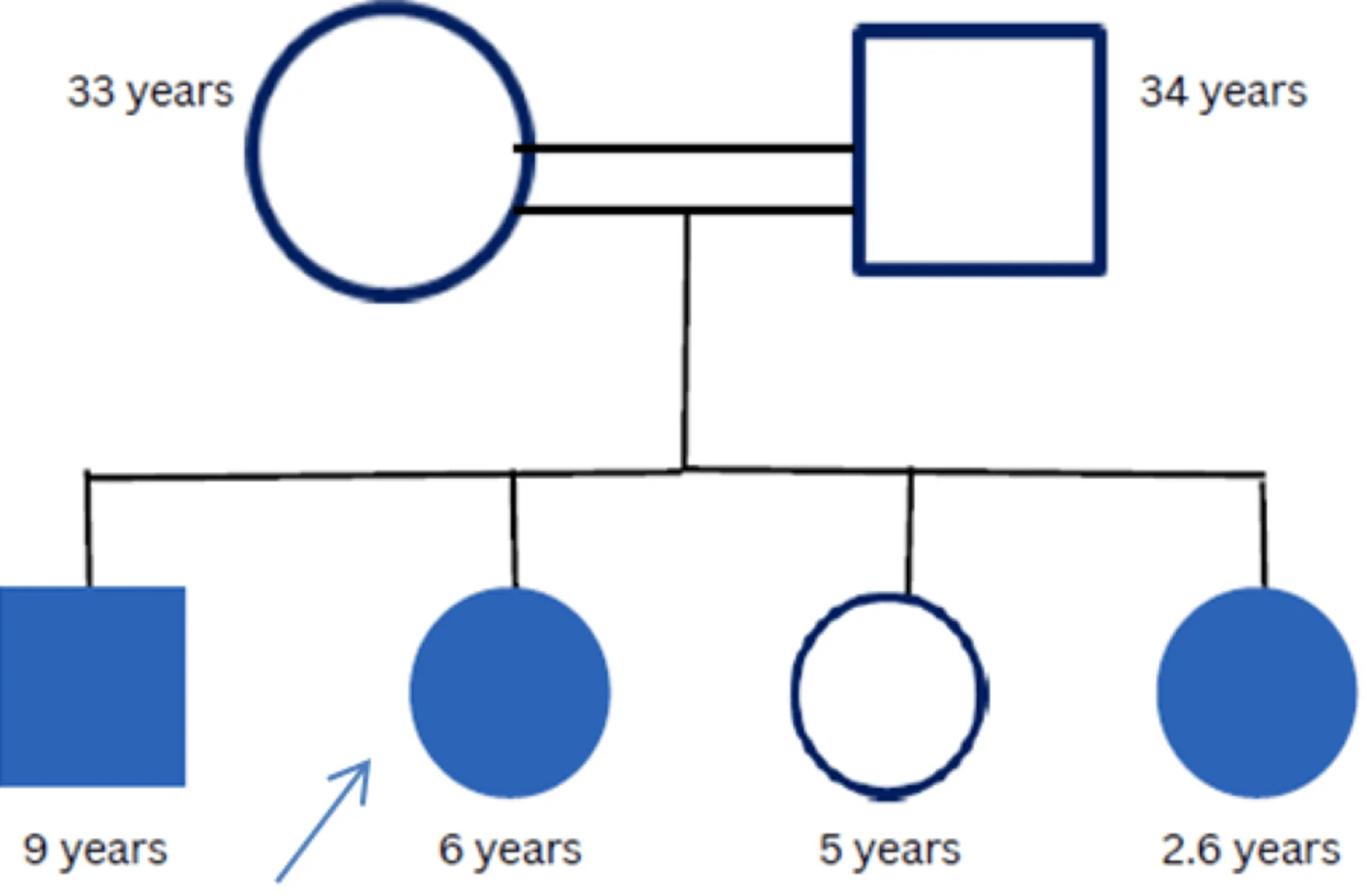
Family pedigree Pedigree of the described family. Open symbols indicate members without PHP1A; filled blue symbols indicate members with PHP1A. The mother has subclinical hypothyroidism consistent with carrier genotype without full AHO phenotype. The arrow denotes the proband. PHP1A: pseudohypoparathyroidism type 1A; AHO: Albright hereditary osteodystrophy

The index patient (proband) was referred from a primary hospital to a secondary hospital, where the other two siblings were recognized to have similar clinical features. Consequently, all three siblings were referred to a tertiary hospital for evaluation by a pediatric endocrinologist. The proband (Figure 1), a six-year-old female patient, presented with painful subcutaneous ossifications causing functional limitation. Similar to her older brother, lesions were first noted at approximately two years of age and progressed over time.

**Figure 2:**
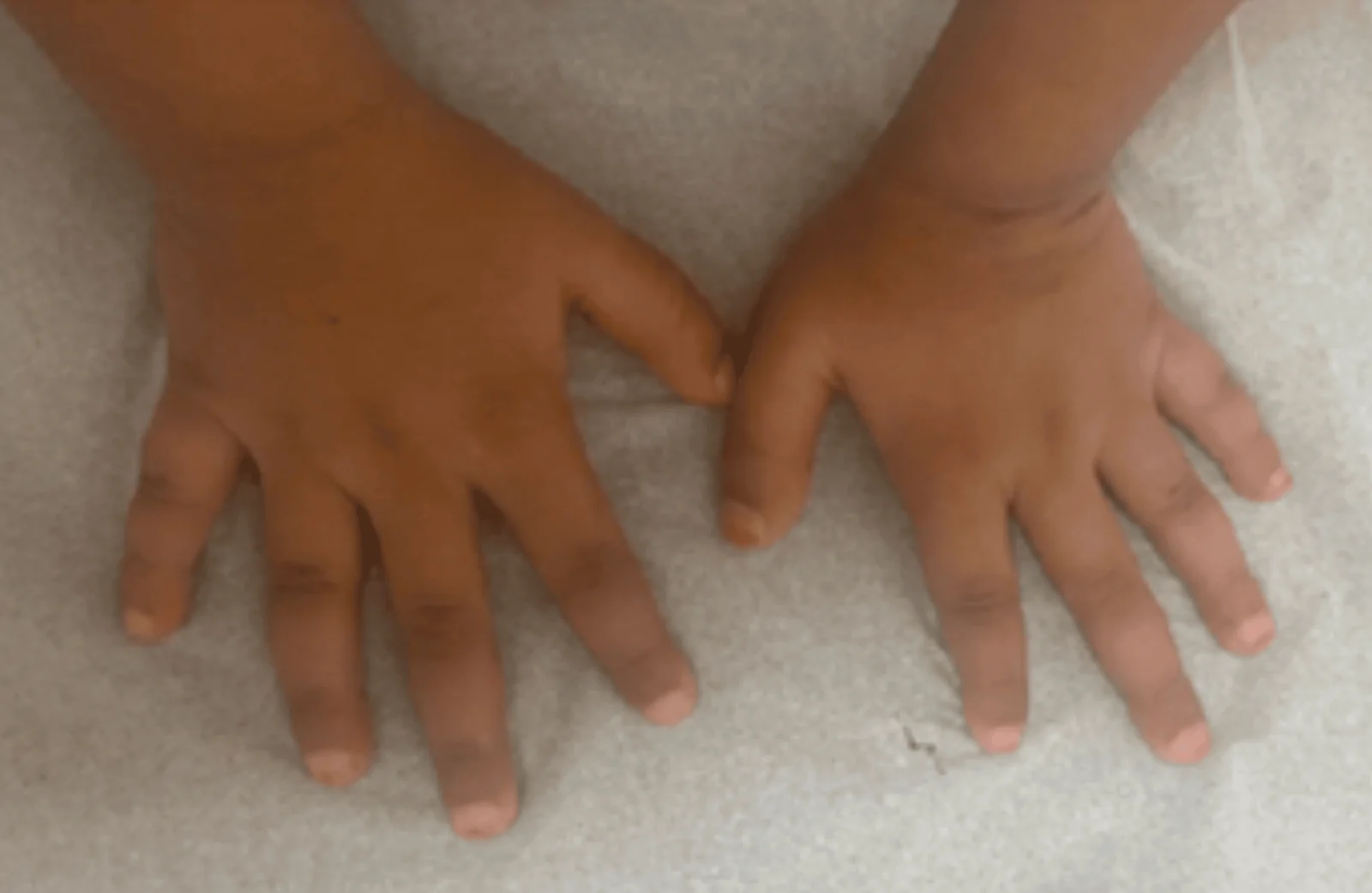
Brachydactyly

Clinical examination demonstrated AHO features including round facies, brachydactyly (Figure 2), and short stature. Growth hormone deficiency was suspected as a contributor to her impaired growth. Soft tissue ossifications were symptomatic and required clinical intervention.

Biochemical evaluation demonstrated marked PTH elevation reaching 39 pmol/L in the presence of normal serum calcium (2.34 mmol/L) and elevated serum phosphate (1.88 mmol/L). TFT revealed subclinical hypothyroidism.

**Figure 3:**
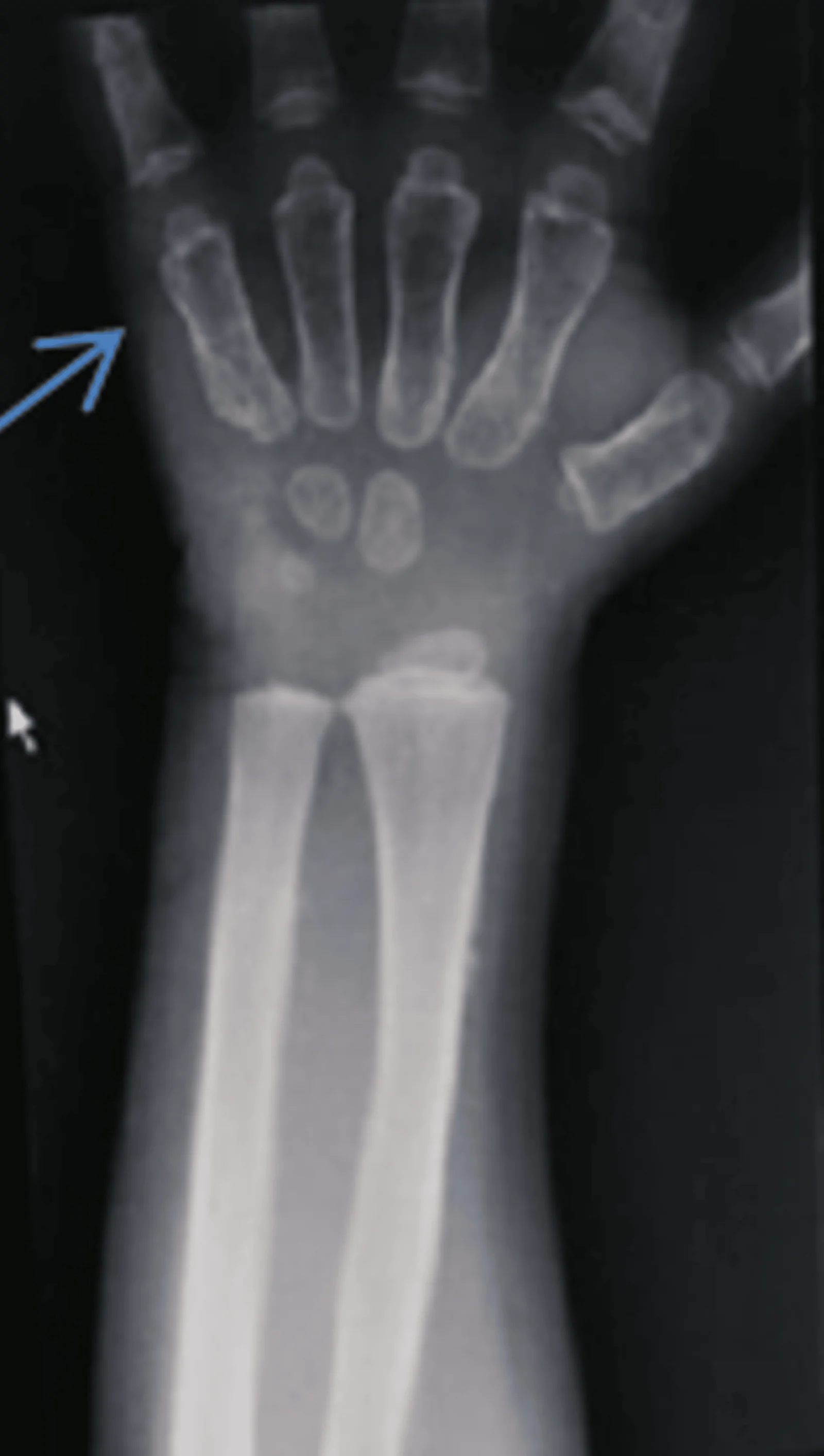
Shortened fourth and fifth metacarpals

Diagnostic investigations included a skeletal survey which demonstrated brachydactyly (Figure 3).

**Figure 4:**
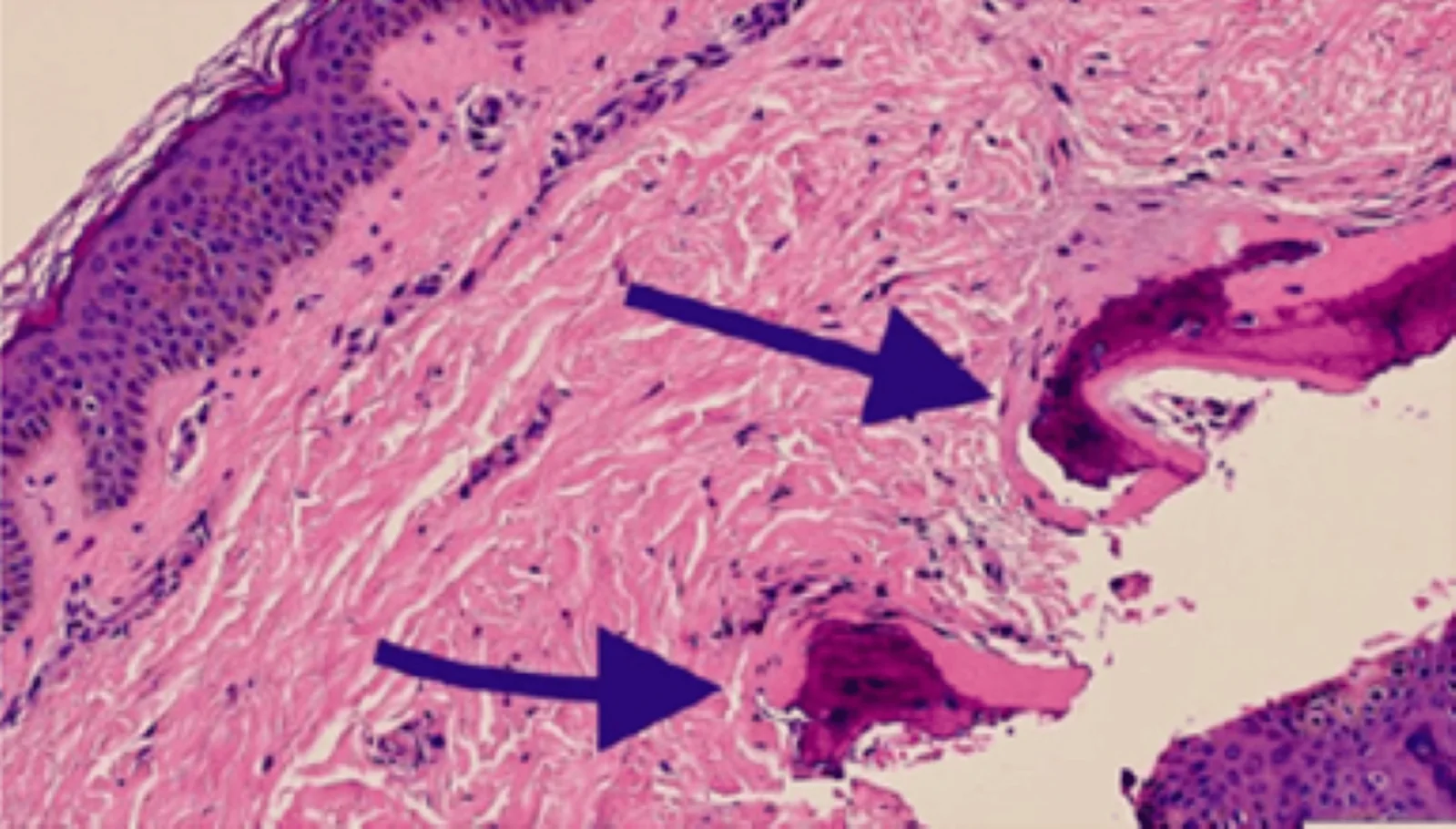
Histopathology of skin biopsy of osteoma cutis The skin biopsy from the thigh showed normal skin with normal epidermis with scanty dermis containing few spicules of trabecular bone (osteoma cutis).

The skin biopsy confirmed osteoma cutis (Figure 4). The cranial computed tomography demonstrated no calcification of the basal ganglia. Genetic analysis identified a heterozygous pathogenic mutation in the GNAS gene, confirming the diagnosis of PHP1A.

Patient 3 (younger sister)

The youngest sibling, a 2.6-year-old female patient, was identified through family screening, clinical and biochemical, at six months of age. At presentation, she was largely asymptomatic.

Clinical examination revealed AHO features including round facies, brachydactyly, and short stature. Small asymptomatic subcutaneous ossifications were present over the abdomen and scalp. Developmental milestones were age-appropriate, and both hearing and vision assessments were normal.

Initial biochemical evaluation demonstrated normal PTH levels (7.2 pmol/L), which progressively increased to 20.8 pmol/L during follow-up for almost five years. Serum calcium remained consistently normal, whereas phosphate levels were mildly elevated. Subclinical hypothyroidism developed during longitudinal follow-up.

Cranial imaging demonstrated no basal ganglia calcification.

Investigations

The differential diagnosis included other forms of PHP (types 1B and 1C), pseudopseudohypoparathyroidism, and progressive osseous heteroplasia. Serial biochemical investigations across all siblings demonstrated the following: progressive elevation of PTH levels, persistent normocalcemia, hyperphosphatemia, mild thyroid-stimulating hormone (TSH) elevation, and normal 25-hydroxyvitamin D concentrations (Table 1).

**Table 1:** Comparative clinical and biochemical characteristics of the family

Parameter	Sibling 1	Sibling 2	Sibling 3	Mother	Father
Age	9 years	6 years	2.6 years	33 years	34 years
Age at presentation	2 years	2 years	8 months	-	-
Primary manifestation	Symptomatic ossifications, cardiac disease	Symptomatic ossifications	Symptomatic ossifications	-	-
Other skeletal manifestations	Moon face, shortened fourth and fifth metacarpal and metatarsal, brachydactyly	Moon face, shortened fourth and fifth metacarpal and metatarsal, brachydactyly	Moon face, shortened fourth and fifth metacarpal and metatarsal, brachydactyly	-	-
Weight	At 50th percentile	At 10th percentile	At 10th percentile	-	-
Height	At 50th percentile	3rd percentile	>3rd percentile	-	-
Parathyroid hormone evolution (range)	Progressive elevation (reach 21 pmol/L)	Progressive elevation (17-39 pmol/L)	Normal → 20.8 (elevated)	Normal	5.9
Adjusted calcium level (1.9-2.7 mmol/L)	2.27	2.34	2.39	2.4	2.19
Serum phosphate (1-1.95 mmol/L)	1.74	1.88	1.96	0.99	1.34
Alkaline phosphatase (90-210 IU/L)	169	195	167	70	67
Vit. D 25 (50-250 nmol/L)	62.7	52.9	70.9	-	-
Vit. D1, 25 (45-137 pg/mL)	170	199	90.6	-	-
Thyroid-stimulating hormone (0.6-4 miIU/L)	6.3	5.89	5.2	7.5	1.23
Free T4 (thyroxine) (11-18 pmol/L)	11.4	13.1	13	10.8	13.1
Thyroid peroxidase antibodies (<60 IU/mL)	39.9	<28	-	>1300	29.7
Extra-skeletal features	Aortic stenosis	Subclinical hypothyroidism	Subclinical hypothyroidism	Subclinical hypothyroidism	No
Symptomatic ossifications	Yes	Yes	No	No	No
Neurocognitive status	Significant impairment (IQ 87)	Significant impairment (IQ 87)	Not tested	Not tested	Not tested
Hearing	Normal	Normal	Normal	Normal	Normal
Vision	Normal	Normal	Normal	Normal	Normal

Genetic analysis performed in one sibling confirmed a heterozygous pathogenic autosomal dominant GNAS mutation, c.2267_2276del p.(Leu756Profs*17). It is a frameshift pathogenic class 1. The inheritance pattern may be consistent with maternal transmission and genomic imprinting; however, parental genetic testing results were not available, and therefore, the parental origin of the variant could not be confirmed.

Diagnosis

The diagnosis of PHP1A was established based on the following: characteristic AHO phenotype, progressive biochemical evidence of PTH resistance, associated TSH resistance, and genetic confirmation of a pathogenic GNAS mutation. The mother had hypothyroidism without overt AHO features, whereas the father was clinically unaffected. The inheritance pattern was consistent with maternal transmission and the known imprinting effects of the GNAS locus. A normal 25(OH)D level excludes vitamin D deficiency as a cause of elevated PTH, supporting PTH resistance consistent with PHP1A while also arguing against autoimmune thyroid disease as an alternative explanation for maternal thyroid dysfunction.

Treatment and follow-up

Management included calcitriol and calcium supplementation when indicated, alongside levothyroxine therapy for hypothyroidism.

Longitudinal follow-up demonstrated progressive elevation of PTH levels in all siblings despite persistent normocalcemia. Ossifications progressed variably, with the eldest sibling developing the most severe functional impairment.

## Discussion

PHP1A demonstrates substantial clinical and biochemical heterogeneity, even among affected members of the same family. This case series illustrates several clinically important features relevant to early recognition and longitudinal management.

Despite a presumed shared genetic background, with one sibling genetically confirmed to have PHP1A and genetic testing for the other two siblings pending, the three siblings demonstrated marked phenotypic heterogeneity, ranging from severe multisystem disease to a near-asymptomatic presentation. Such variability likely reflects the influence of epigenetic mechanisms, tissue-specific imprinting, and additional genetic or environmental modifiers [6].

An important observation in this family was the prolonged phase of compensated PTH resistance. All siblings demonstrated persistent normocalcemia despite progressive PTH elevation and hyperphosphatemia. This finding challenges rigid reliance on classical biochemical diagnostic criteria in children and supports the concept that endocrine resistance in PHP1A evolves gradually over time [3,7,8].

The presence of congenital aortic stenosis in the eldest sibling may broaden the recognized cardiovascular spectrum associated with GNAS-related disorders. Although cardiovascular anomalies are not traditionally emphasized in PHP1A, emerging literature suggests potential associations between GNAS dysfunction and structural cardiac abnormalities [9].

Neurocognitive involvement also varied considerably among siblings. One child demonstrated significant learning difficulties, whereas the youngest sibling maintained age-appropriate developmental milestones. This observation is consistent with reports indicating variable central nervous system involvement in PHP1A [10].

The burden and distribution of ectopic ossifications differed substantially within the family, emphasizing the variable expression of osteoma cutis even among genetically related individuals [11]. Notably, none of the siblings demonstrated basal ganglia calcification, which may help differentiate PHP1A from other calcium-phosphate disorders.

Learning points

PHP1A results from maternally inherited inactivating mutations in the GNAS gene. Features of AHO may precede overt biochemical abnormalities. Compensated PTH resistance with normocalcemia can persist for years in pediatric patients. Significant intrafamilial phenotypic heterogeneity may occur despite a shared genetic background. Longitudinal biochemical surveillance is essential for early detection of evolving hormone resistance.

## Conclusions

This familial case series shows that PHP1A presents with considerable variability and that physical signs of AHO often appear years before the typical biochemical abnormalities.

Because hormone resistance can be initially compensated and later progress to overt endocrine dysfunction, ongoing longitudinal monitoring is essential; early recognition of AHO features, family screening, and genetic confirmation enable timely diagnosis and facilitate planned multidisciplinary monitoring and targeted intervention.
